# P-129. Evaluation of the Mannheim Peritonitis Index, the Neutrophil/Lymphocyte Ratio, and the Platelet/Lymphocyte Ratio as Predictive Parameters of Clinical Outcomes of Patients with Intra-Abdominal Infection in a Teaching Hospital in Nicaragua

**DOI:** 10.1093/ofid/ofae631.334

**Published:** 2025-01-29

**Authors:** Freda L Alvarado-Malueño, Guillermo D Porras-Cortés

**Affiliations:** Hospital Dr. Fernando Vélez Paiz, Managua, Managua, Nicaragua; Hospital Dr. Fernando Vélez Paiz, Managua, Managua, Nicaragua

## Abstract

**Background:**

Intra-abdominal infections are associated worldwide with relatively high mortality. Decisions in patient management and clinical outcomes may be determined by specific risk factors that can be assessed in the form of scores or indices, and ratios. The objective of this study was to evaluate the Mannheim Peritonitis Index (MPI), the Neutrophil/Lymphocyte Ratio (NLR), and the Platelet/Lymphocyte Ratio (PLR) as predictors of mortality and other clinical outcomes in this type of patients at a teaching and referral hospital (Dr. Fernando Vélez Paiz Hospital) in Managua, Nicaragua.

**Table 1.**
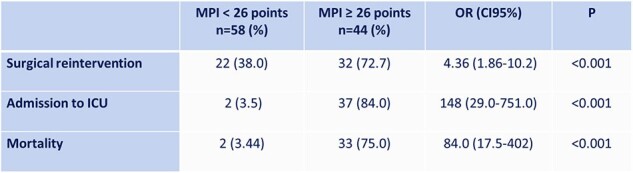
Mannheim Peritonitis Index and Surgical Reintervention, Admission to ICU, and Mortality

**Methods:**

This is a retrospective, cross-sectional, analytical study. Patients hospitalized for complicated intra-abdominal infection (cIAI)an between November 2021 and November 2023 were included. The relationship of MPI, NLR, and PLR with mortality and other clinical outcomes as need for reoperation, requiring admission to intensive care unit, and length of hospital stay was analyzed. The cut-off points of previous reference studies for MPI (≥ 26 points), NLR (≥ 11), and PLR (≥ 222) were used. Sensitivity, specificity and a ROC curve (AUC) were calculated for each of the predictive instruments evaluated

**Table 2.**
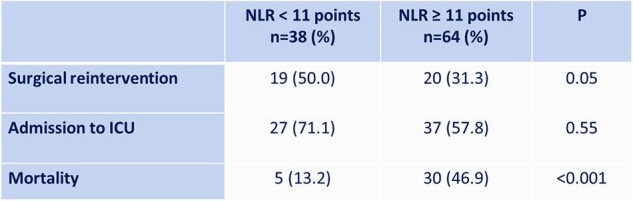
Neutrophiles/Lymphocytes Ratio and Surgical Reintervention, Admission to ICU, and Mortality

**Results:**

102 patients with cIAI were included in the study. The mean age was 56.05 ± 21.25 years-old. The most common etiology was acute appendicitis (39.2%). Overall mortality was 34.3%. The need for surgical reintervention was observed in 72.7% of patients with MPI ≥ 26 points, 31.3% of patients with NLR ≥ 11, and 45.0% of patients with PLR ≥ 222. Regarding requiring admission to the intensive care unit, it was observed in 84.0% of patients with MPI ≥ 26 points, 57.8% of patients with NLR ≥ 11, and 27.5% of patients with PLR ≥ 222. Mortality in patients with MPI ≥ 26 points was 75.0%, and 46.9% in patients with NLR ≥ 11 (Table 1 and 2). Theand specificity of MPI for mortality was 94.3% and 47.9% respectively, with an AUC of 0.95. The sensitivity and specificity of NLR for mortality were 85.7% and 49.3% respectively (Table 3).

**Table 3.**
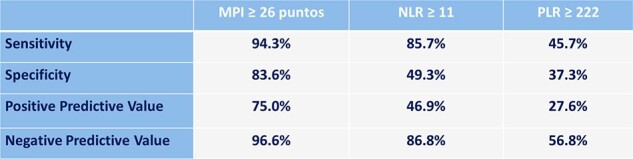
Sensitivity and Specificity of MPI and NLR for Mortality Prediction.

**Conclusion:**

The MPI ≥ 26 points predicts mortality, a higher probability of surgical reoperation, ICU admission, and prolonged hospital stay. In the population of this study, the cut-off point with the best diagnostic reliability was MPI ≥ 25 points. The NLR ≥ 11 points is also a significant predictor of mortality.

**Disclosures:**

**All Authors**: No reported disclosures

